# Subjective Interactions of Wood-Derived Olfactory and Visual Stimuli During Work and Rest

**DOI:** 10.3390/ijerph22050724

**Published:** 2025-05-02

**Authors:** Takashi Shima, Katsuhiko Sakata, Yuko Tsunetsugu

**Affiliations:** 1 Kajima Technical Research Institute, Kajima Corporation, 2-19-1, Tobitakyu, Chofu-shi 182-0036, Japan; sakatak@kajima.com; 2 Department of Biomaterial Sciences, Graduate School of Agricultural and Life Sciences, The University of Tokyo, 1-1-1, Yayoi, Bunkyo-ku 113-8657, Japan

**Keywords:** wood-derived stimuli, impression, mood states, fatigue, workplace design

## Abstract

This study investigated the effects of wood-derived visual and olfactory stimuli on subjective evaluations during work and rest. Twelve participants experienced seven conditions involving walnut panel interiors, Hinoki cypress scents, or their combination, introduced during either work or rest phases. A within-subjects design was used to assess spatial impressions, mood states, and fatigue. Visual stimuli significantly improved visual impressions across items such as “settling–unsettling” and “luxurious–simple” (*p* < 0.05). The Hinoki scent enhanced olfactory impressions and, when introduced during rest, reduced negative mood states including “Anger–Hostility” and “Confusion–Bewilderment” (*p* < 0.05). Combined stimuli further improved scent impressions (*p* < 0.1), suggesting cross-modal effects. However, their introduction during work increased “Anger–Hostility” (*p* < 0.05), highlighting the importance of context. Although the within-subjects design supports internal validity, the small sample size (*n* = 12) limits generalizability. These findings provide insight into the context-sensitive use of wood-derived sensory elements in workplace design.

## 1. Introduction

In recent years, growing attention has been paid to employee health and well-being in the workplace. Numerous studies have reported that occupational stress reduces productivity and adversely affects both mental and physical health [1,2]. In Japan, interest in improving the work environment has increased since the revision of the Labor Standards Act and the enactment of the Work Style Reform Promotion Act. However, a 2021 survey by the Ministry of Health, Labour and Welfare indicated that 53.3% of employees still experience high levels of anxiety or stress in the workplace [3], emphasizing the ongoing need for stress mitigation strategies.

One effective approach is the introduction of natural elements into architectural design. Kaplan’s Attention Restoration Theory (ART) [4] proposes that natural environments promote psychological recovery through four features: fascination, being away, extent, and compatibility. While incorporating greenery is common, the use of wood in interiors has recently gained attention not only for its restorative psychological effects but also for its environmental benefits, such as reducing CO_2_ emissions and storing carbon [5].

Previous research has examined the psychological effects of wood through multiple sensory modalities. In olfactory studies for example, Miyazaki et al. [6] demonstrated the restorative potential of Taiwanese cypress essential oil, while Chen et al. [7] found that inhalation of Hinoki and Menki essential oils improved mood and reduced sympathetic activity. Matsubara et al. [8] showed that volatile organic compounds (VOCs) emitted by cedar influenced salivary amylase activity, a stress biomarker, and enhanced odor impressions. Additionally, compounds such as α-pinene and D-limonene were found to influence heart rate and subjective mood [9,10]. However, inappropriate concentration levels of such compounds may result in unpleasant impressions [11], emphasizing the need for careful control of dosage and delivery methods, such as natural volatilization and essential oil diffusers [12].

Visual stimuli from wood have also been found to evoke positive emotional and physiological responses. Tsunetsugu et al. [13] observed changes in subjective impressions and autonomic nervous activity based on the proportion of oak used in interiors. Burnard et al. [14] demonstrated that wooden furniture in offices affected salivary cortisol levels. More recently, researchers have explored factors such as wood grain direction and knots [15,16], as well as immersive presentation methods using virtual reality (VR) [17].

Importantly, several studies have examined the interaction of sensory modalities. Demattè et al. [18] showed that individuals with a stronger affinity for nature were more sensitive to subtle olfactory and tactile cues in wooden environments. Nakashima et al. [19] reported that visual responses intensified when cedar scent was present. Kim et al. [20] found that participants perceived more warmth when viewing VR representations of wood interiors than non-wooden ones. These findings suggest a mutual influence among olfactory, visual, and air-quality perceptions.

Efforts to apply these findings in real-world environments are increasing. Field studies, such as Whyte et al. [21], have examined wooden interiors in office settings. However, due to complex contextual factors—such as interpersonal dynamics and task-related stress—generalizing effects remains challenging. As a bridge between laboratory and field research, model experiments have emerged. For example, Zhang [22], Matsubara [8], and Shima [23] implemented protocols involving work tasks and rest intervals in controlled environments to assess the restorative effects of wood-derived sensory stimuli. Zhang et al. highlighted recovery during rest phases, Matsubara et al. demonstrated olfactory-induced stress reduction, and Shima et al. reported that Hinoki scent prevented mood deterioration.

Despite these advances, several gaps remain. For instance, the effects of visual stimuli alone during rest are unclear. Moreover, the mechanism by which Hinoki scent suppresses stress—whether during work or rest—remains to be clarified. Further exploration of the combined and isolated effects of olfactory and visual stimuli is warranted.

This study tested the following hypotheses:

1. The introduction of wood-derived visual and olfactory stimuli suppresses increases in psychological stress during work.
2. The introduction of these stimuli enhances stress recovery during rest periods following work.
3. Subjective evaluations of olfactory and visual impressions differ when stimuli are introduced in combination, compared to when presented individually.

By verifying these hypotheses, this study aims to clarify the behavioral effects of wood-based sensory environments and provide guidance for effective environmental design in office spaces.

## 2. Materials and Methods

### 2.1. Participants

Twelve adult participants (six males and six females) were recruited for this study, comprising three males and three females in their 30s, and three males and three females in their 40s. The participants had a mean age of 40.8 ± 6.3 years (range: 30–48 years). All participants were confirmed to be nonsmokers and had no history of rhinitis or color vision deficiency. They were also instructed to abstain from alcohol consumption on the day prior to each experimental session. All procedures were conducted in accordance with the ethical guidelines approved by the Ethics Committee of Kajima Technical Research Institute.

### 2.2. Study Protocol

The experiment was conducted over seven days, during which all participants experienced each of the seven experimental conditions. To reduce carryover effects, a minimum interval of two days was scheduled between sessions. Each session was conducted on alternate days for each participant.

**Figure 1:**
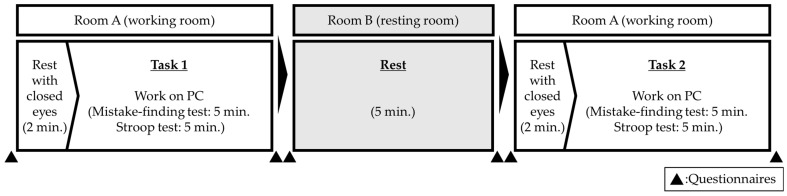
Experimental procedure.

During each session, participants entered a temperature- and humidity-controlled experimental room (23–26 °C, 40–60% RH) with approximately 500 lux of illuminance at head level and were seated in front of a PC. After a 2 min eye-closing period to stabilize their mental state, participants completed a 10 min task (Task 1). They then moved to a separate resting room for a 5 min break (rest), returned to the original room, repeated the 2 min eye-closing procedure, and completed a second 10 min task (Task 2). Subjective evaluation questionnaires were administered after entering and before leaving the working or resting rooms (Figure 1).

The combinations of stimuli used in the experiment are summarized in Table 1. These conditions varied in the presence or absence of olfactory (Hinoki essential oil) and visual (walnut panel interiors) stimuli, as well as in the timing of stimulus introduction—during either the task or rest phase. For clarity, each condition is labeled as follows: NN (no stimuli), HN-T (olfactory stimulus during task), HN-R (olfactory stimulus during rest), NW-T (visual stimulus during task), NW-R (visual stimulus during rest), HW-T (both stimuli during task), and HW-R (both stimuli during rest).

The three conditions that did not involve olfactory stimuli (NN, NW-T, NW-R) were randomly assigned to Days 2, 4, and 6, ensuring that each of these days included one of the non-olfactory conditions. Meanwhile, the four conditions involving olfactory stimuli (HN-T, HN-R, HW-T, HW-R) were randomly allocated across Days 1, 3, 5, and 7. In addition, olfactory conditions were scheduled non-consecutively to avoid potential sensory adaptation or fatigue.

Few previous studies have attempted to independently introduce wood-derived visual and olfactory stimuli in a controlled experimental setting. In the present study, conditions without olfactory stimuli were implemented by using a thin (~0.5 mm) wood veneer for visual presentation in order to minimize the release of natural wood scents. Conversely, conditions without visual stimuli were implemented by using only an aroma diffuser without any visible wood materials, ensuring no visual cues were present. This approach enabled strict separation of unimodal and combined sensory stimuli.

Additionally, the durations of the task and rest phases were designed to align with previous experimental studies. Specifically, the 10 min task and 5 min rest periods were adopted to replicate the timing and structure reported by Zhang et al. [22] and Shima et al. [23].

### 2.3. Olfactory Stimuli

Hinoki cypress essential oil (Tree of Life Co., Ltd., Tokyo, Japan) was used as the olfactory stimulus. The scent was diffused into the experimental room using an aroma diffuser from the same manufacturer (Model No. 08-802-9240). The essential oil was diluted at a ratio of 1 mL of oil to 10 mL of water. In the control condition without olfactory stimuli, only water was used in the diffuser.

To ensure that the scent intensity did not cause discomfort, a preliminary study was conducted with 12 participants in their 20s to determine an appropriate concentration. In this pre-test, participants entered a room where the Hinoki scent was diffused, performed ten deep inhalations, and rated the odor intensity using a questionnaire. Based on the results, the concentration used in the main experiment corresponded to a range described as “weak” to “easily noticeable”.

To further control for sensory comfort and standardize exposure levels, air samples were collected at nose height prior to each session, and the concentration of α-pinene—the primary volatile compound in Hinoki oil—was analyzed using gas chromatography–mass spectrometry (GCMS QP2020NX; Shimadzu Corporation, Kyoto, Japan). Referring to previous research indicating that concentrations exceeding 14.3 mg/L could be perceived as “slightly unpleasant” [24], the α-pinene level was adjusted to remain below this threshold. The final concentration ensured a detectable but comfortable olfactory experience for participants.

### 2.4. Visual Stimuli

**Figure 2:**
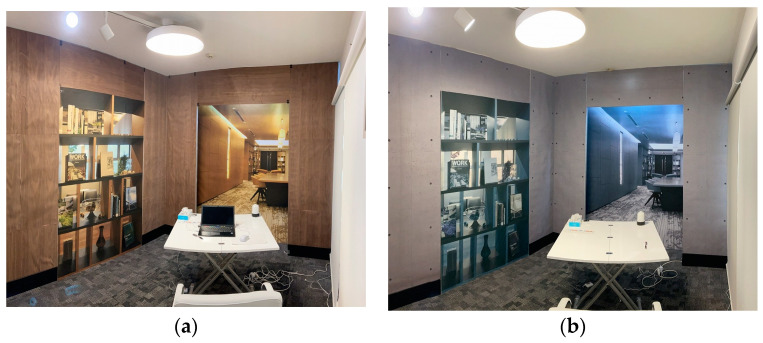
Visual conditions of experimental rooms. (**a**) Wings and hanging walls were covered with sliced veneers of walnut panels (each panel’s size was 950 mm × 450 mm). (**b**) Wings and hanging walls were covered with concrete-looking wallpaper.

Two identical experimental rooms (each approximately 10 m^2^ in area) were constructed for this study. In the visual stimulation conditions, walnut wood panels were installed on the ceilings and walls (Figure 2). In the control condition, concrete-looking wallpaper was used instead of wood panels. To simulate the appearance of a larger office environment and enhance spatial realism, large photographs of a training center with a wood-textured interior were affixed to the front and right walls in both rooms. In the control room, the same images were presented in grayscale to minimize visual warmth and naturalness.

A preliminary study was conducted to select the wood species for visual stimulation. Ten participants evaluated three types of wood panel samples based on their visual impression, using a 7-point Likert scale ranging from −3 (very negative) to +3 (very positive). Among the tested samples, walnut received the highest average rating and was thus selected for use in the main experiment. The arrangement and design of visual stimuli aimed to control both naturalness and spaciousness, while maintaining a consistent visual environment across conditions.

### 2.5. PC Tasks

Each experimental session required participants to complete two cognitive tasks on a personal computer, designed to simulate work-related stress. The first task was a mistake-finding test conducted for five minutes. Participants were shown two versions of a text slip side-by-side on the monitor and were instructed to identify differences in characters between the two versions. The second task was a Stroop test, also lasting five minutes. In this task, participants were presented with one word per second on the screen, where each word was the name of a color (“red”, “blue”, “yellow”, or “green”) written in one of the four ink colors. There were 16 possible combinations of word meaning and text color. Participants were instructed to press the key corresponding to the color of the word, not the word itself. Although originally developed to assess selective attention [25], the Stroop test was implemented in this study as a stress-inducing task due to its cognitive demand. These two tasks (mistake-finding and Stroop) were administered consecutively during both Task 1 and Task 2 sessions, immediately following each 2 min eyes-closed preparation period.

### 2.6. Measurement

Three questionnaires for subjective evaluation were administered before and after the participants entered the room. Subjective ratings were provided on a seven-point scale to describe the effects of the olfactory or visual impressions. The olfactory impression was evaluated using six items, which are “good-bad”, “like-dislike”, “familiar-unfamiliar”, “settling-unsettling”, “luxurious-simple”, and “mild-pungent”. The visual impression was evaluated using six items, which were “like-dislike”, “familiar-unfamiliar”, “settling-unsettling”, “luxurious-simple”, “spacious-small”, and “natural-artificial”.

The Japanese version of the Profile of Mood States 2nd Edition Short Form (POMS2; Kaneko Shobo Co., Tokyo, Japan [26]) was administered to assess negative mood states. The POMS2 evaluates the state of mood and emotion using seven scales: “Anger-Hostility”, “Confusion-Bewilderment”, “Depression-Dejection”, “Fatigue-Inertia”, “Tension-Anxiety”, “Vigor-Activity”, and “Friendliness”. There were five questions on each scale, and each question was evaluated on a scale of 0 to 4, representing participants’ mood. The Total Mood Disturbance (TMD) score, indicating a comprehensive negative mood, is calculated by the scores of all scales except “Friendliness”.

The Jikaku-sho Shirabe (Industrial Fatigue Research Committee of Japan [27]) was used to measure subjective fatigue. This tool provides a subjective evaluation of fatigue on five scales: “Drowsiness”, “Instability”, “Uneasiness”, “Dullness”, and “Eyestrain”. Each dimension comprised five questions, which the participants answered using a five-point scale from 1 to 5. By observing changes in scores over time, the extent of the participants’ fatigue complaints can be assessed.

The selection of the POMS2 and Jikaku-sho Shirabe scales followed established usage in Japanese occupational and stress-related research, both of which have been psychometrically validated in prior studies.

### 2.7. Statistical Analysis

All statistical analyses were performed using SPSS (version 28.0.1.1; IBM Corp., Armonk, NY, USA). The significance level was set at 5%, and trends toward significance were considered at the 10% level. For impression ratings, scores obtained after Task 1, the rest period, and Task 2 were compared across experimental conditions. Visual impression scores were compared among the NN, NW-T, and HW-T conditions after Tasks 1 and 2, and among the NN, NW-R, and HW-R conditions after the rest period. Similarly, olfactory impression scores were compared among the NN, HN-T, and HW-T conditions after Tasks 1 and 2, and among the NN, HN-R, and HW-R conditions after the rest period.

To evaluate the effects of wood-derived olfactory and visual stimuli on subjective stress during work tasks, changes in mood states and subjective fatigue scores were calculated. The change during Task 1 was defined as the score after Task 1 minus the baseline score obtained before the session, while the change during Task 2 was calculated by subtracting the score after the rest period from the score obtained after Task 2. These score changes were compared among four conditions: a no-stimulus condition (NN), single-stimulus conditions (HN-T and NW-T), and a combined-stimulus condition (HW-T).

In addition, to assess the effects of stimuli introduced during the rest period, changes in mood states and fatigue scores were calculated as the difference between the scores after and before the rest period. These differences were compared among the no-stimulus condition (NN), single-stimulus conditions (HN-R and NW-R), and the combined-stimulus condition (HW-R).

The normality of all score difference data was tested using the Shapiro–Wilk test, which indicated significant deviation from a normal distribution (*p* < 0.05). Accordingly, the Friedman test was used to evaluate differences among conditions. For items showing a trend toward significance (*p* < 0.10) in the Friedman test, post hoc comparisons were conducted using the Wilcoxon signed-rank test with Holm correction.

## 3. Results

### 3.1. Impression Evaluation

Table 2 presents the results of olfactory impression ratings, while Table 3 shows the results of visual impression ratings. Table 2 includes the mean scores and statistical comparisons for each condition: the control condition (NN), the Hinoki scent-only condition, and the combined condition in which both Hinoki scent and walnut wall panels were introduced. Table 3 displays the corresponding results for visual stimuli, including the control condition, the walnut panel-only condition, and the combined condition.

For olfactory impressions, the NN condition received significantly lower ratings than both the Hinoki-only and the combined conditions across all time points for the items “good–bad”, “like–dislike”, “luxurious–simple”, and “intensity”. After Task 2, scores for the items “settling–unsettling” and “mild–pungent” were also significantly lower under the NN condition compared to the other two. Additionally, after the rest period, the NN condition had significantly lower ratings on “mild–pungent” compared to the combined condition, and on “familiar–unfamiliar” compared to the Hinoki-only condition. These findings suggest that the introduction of Hinoki scent consistently enhanced olfactory impressions across all spatial settings. Interestingly, the item “good–bad” showed significantly lower ratings in the Hinoki-only condition than in the combined condition after Task 1, and “luxurious–simple” also showed significantly lower scores in the Hinoki-only condition than in the combined condition after Task 2. This indicates a possible interaction effect, in which visual stimuli influence the perception of scent quality.

For visual impressions, the NN condition was rated significantly lower than both the walnut-only and the combined conditions across all time points on the “like–dislike” scale. After Tasks 1 and 2, the NN condition also received significantly lower scores on “luxurious–simple” and “natural–artificial”. After the rest period, the NN condition remained significantly lower on “luxurious–simple”. On the “settling–unsettling” scale, the NN condition was significantly lower only after Task 1. These findings indicate that the walnut interior significantly improved visual impressions compared to the concrete-looking interior across various dimensions. However, unlike olfactory impressions, no significant differences were observed between the single and combined stimulus conditions for visual impressions.

### 3.2. Mood States and Subjective Fatigue After the Tasks

**Figure 3:**
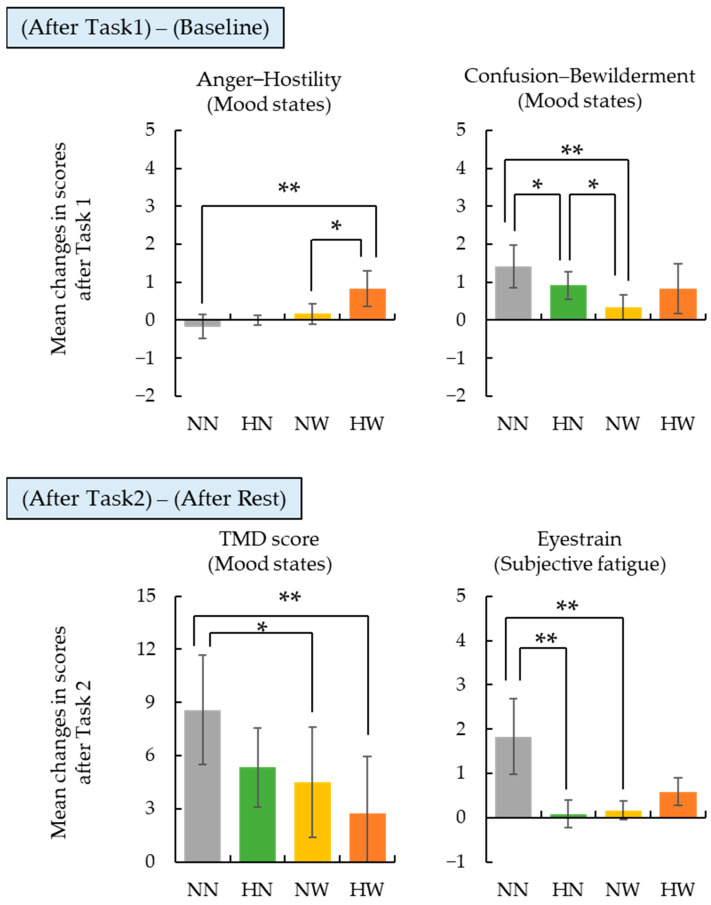
Changes in mood and fatigue scores after Task 1 and Task 2 under different stimulation conditions. Bar graphs show the mean changes in scores (± SE) for “Anger–Hostility”, “Confusion–Bewilderment”, “Total Mood Disturbance (TMD)”, and “Eyestrain”. Statistically significant differences among conditions were identified by Wilcoxon signed-rank tests with Holm correction (* *p* < 0.1, ** *p* < 0.05).

To examine the effects of wood-derived olfactory and visual stimuli introduced during work sessions, we compared changes in mood states and subjective fatigue across stimulus conditions, including the control condition (NN). Results from the Friedman test showed significant differences among conditions for “Anger–Hostility” (*p* = 0.044) and “Confusion–Bewilderment” (*p* = 0.091) after Task 1, and for Total Mood Disturbance (TMD; *p* = 0.026) and “Eyestrain” (*p* = 0.035) after Task 2. The change scores for these scales are presented in Figure 3.

For “Anger–Hostility” after Task 1, the HW condition showed a significantly greater increase in scores compared to both the NN and NW conditions. For “Confusion–Bewilderment”, the NN condition exhibited a significantly greater increase than the NW and HN conditions, and the HN condition showed a significantly greater increase than NW. After Task 2, the increase in TMD scores under the NN condition was significantly greater than under the HN and NW conditions. Similarly, for “Eyestrain”, the increase was significantly greater in the NN condition than in both HN and NW, suggesting that sensory stimuli contributed to stress suppression and eye comfort during tasks.

### 3.3. Mood States and Subjective Fatigue After Rest

**Figure 4:**
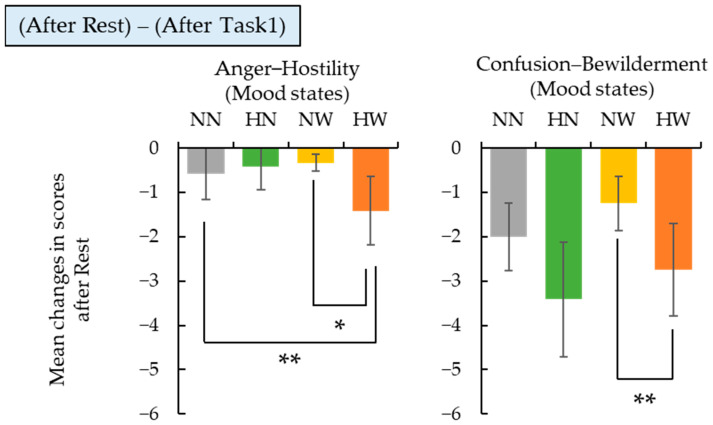
Changes in mood scores during rest and subsequent tasks under different stimulation conditions. Bar graphs show the mean changes in scores (± SE) for “Anger–Hostility” and “Confusion–Bewilderment”. Statistically significant differences among conditions were identified by Wilcoxon signed-rank tests with Holm correction (* *p* < 0.1, ** *p* < 0.05).

To investigate the impact of wood-derived sensory stimuli introduced during rest periods, we compared changes in mood and subjective fatigue across stimulus conditions, including the control (NN). The Friedman test revealed marginally significant differences among conditions for “Anger–Hostility” (*p* = 0.058) and “Confusion–Bewilderment” (*p* = 0.079). Figure 4 presents the change scores for these scales.

For “Anger–Hostility”, the HN condition showed a significantly greater decrease in scores compared to the NN and NW conditions. For “Confusion–Bewilderment”, the HW condition showed a significantly greater decrease than the NW condition. These results suggest that the introduction of Hinoki scent and the combination of Hinoki scent and wood visuals during rest may promote emotional recovery and reduce cognitive disorientation more effectively than visual stimulation alone.

## 4. Discussion

This study investigated the effects of wood-derived olfactory (Hinoki scent) and visual (walnut panel interior) stimuli on subjective impressions, mood states, and fatigue during both work and rest phases. While previous research has explored the psychological benefits of wood-based sensory stimuli, few studies have systematically compared their individual and combined effects under varying behavioral contexts such as task engagement and relaxation.

The impression ratings indicated that both visual and olfactory stimuli significantly improved spatial impressions compared to the control condition, consistent with findings by Tsunetsugu et al. (oak interiors) [13], Sakuragawa et al. (Hinoki interiors) [28], and Chen et al. (essential oil inhalation) [7]. Furthermore, this study contributes novel evidence that walnut-derived visual stimuli, which have received limited attention, can enhance spatial impression relative to concrete-like interiors.

A notable finding was that the combined condition resulted in higher olfactory impression scores than the olfactory-only condition for certain items (e.g., “good–bad”, “luxurious–simple”). This suggests a cross-modal interaction in which visual stimuli modulate olfactory perception, aligning with prior research by Demattè et al. [18] and Nakashima et al. [19]. The visual dominance theory proposed by Rock et al. [29] further supports the idea that visual stimuli strongly influence the interpretation of other sensory inputs, which may explain why the presence of walnut visuals reinforced the positive evaluation of Hinoki scent.

However, mood-related outcomes were more complex. For instance, after Task 1, “Anger–Hostility” scores were significantly higher in the combined condition compared to both the no-stimulus and visual-only conditions, suggesting that combined stimuli do not necessarily enhance emotional well-being. This finding may be explained by the “attenuation effect” proposed by Qi et al. [30], where a strong sensory input reduces the effectiveness of other stimuli. In cognitively demanding contexts such as task performance, the combination of multiple stimuli may have led to sensory overload, exacerbating stress responses instead of alleviating them.

Temporal differences were also observed in sensory evaluations. During the task phase, olfactory impressions tended to be lower, potentially due to attentional constraints. In contrast, during rest, participants may have been more attuned to sensory input, allowing for more accurate evaluations. Consistent with this, mood recovery effects were more pronounced after rest, particularly for “Confusion–Bewilderment” and “Anger–Hostility”, where both olfactory and combined stimuli led to significant score reductions.

These results highlight the importance of integrated decision-making in multisensory environmental design. Combined stimuli do not always produce additive effects; rather, the interaction between sensory modalities and contextual factors must be considered. Practically, visual stimuli such as wood interiors may be most effective during active work, while olfactory stimuli may yield stronger restorative effects when introduced during rest. In office design, this could translate into permanent wood installations in task areas and timed scent delivery in relaxation zones. Personalized scent control systems could also enhance perceived autonomy and satisfaction.

In addition to these sensory-specific findings, it is important to consider how wood-derived stimuli integrate with broader frameworks used in building performance evaluation. Recent studies emphasize the importance of Indoor Environmental Quality (IEQ), which includes factors such as air quality, thermal comfort, acoustic environment, and lighting, alongside psychological and physiological parameters [31,32]. Material emissions, including volatile organic compounds (VOCs) from interior finishes, have also been linked to Sick Building Syndrome (SBS), as reported by Subri et al. (2024) [33]. Moreover, Coulby et al. [34] have proposed comprehensive IEQ monitoring systems that combine objective metrics and subjective evaluations. These are also consistent with recent research [35] on real-time environmental sensing and occupant-centered adaptive design.

In this context, future studies on wood-derived stimuli should extend beyond sensory evaluations to include interactions with ventilation strategies, thermal regulation, and chemical emissions. A truly human-centered approach to interior design requires the integration of both physiological and perceptual feedback, in alignment with current international standards in IEQ research.

## 5. Conclusions

This study examined the psychological effects of wood-derived olfactory (Hinoki scent) and visual (walnut panel) stimuli introduced during work or rest phases. The findings revealed that both types of stimuli significantly enhanced spatial impressions compared to the control condition. Notably, the walnut panel interior, which has been less frequently investigated in prior research, showed a clear improvement in impression ratings compared to concrete-like interiors, indicating its potential as a valuable design material.

Additionally, in the combined stimulus condition, olfactory impressions were more positively evaluated than in the olfactory-only condition for some items. This suggests the presence of cross-modal effects, in which visual input modulates olfactory perception, aligning with previous research on sensory integration. These results imply that the integrated design of multiple sensory inputs may enhance spatial evaluations more effectively than single-modal approaches.

However, results related to mood and fatigue were more complex. In some conditions, especially when combined stimuli were introduced during task phases, negative emotional responses such as increased “Anger–Hostility” were observed. These findings highlight that sensory stimuli do not always produce uniformly positive effects and that their psychological impact may vary depending on the combination, context, and timing of introduction.

Based on these results, it is suggested that visual stimuli may be more suitable for enhancing impressions and maintaining concentration during task phases, while olfactory stimuli may be more effective in promoting emotional recovery during rest. For practical applications, it is recommended that permanent wood visual elements be integrated into workspaces, while adjustable olfactory stimuli be selectively introduced in relaxation zones or short-term occupancy areas. In environments such as individual booths, where work and rest alternate repeatedly, strategic switching and combining of sensory inputs may be particularly effective.

This study has several limitations. First, the sample size was limited to 12 participants, which necessitates cautious interpretation of generalizability. However, previous studies investigating the psychological and physiological effects of wood-derived stimuli (e.g., by Kimura et al. [36] and Matsubara et al. [8]) have reported meaningful findings using similar sample sizes. Furthermore, this study adopted a complete within-subjects design, in which all participants experienced all seven stimulus conditions. This experimental structure minimized the influence of individual differences and allowed for sensitive comparisons between conditions, partially compensating for the limited sample size.

Second, only one type of wood species (walnut and Hinoki) and one scent intensity level were used. Therefore, the effects of species-specific characteristics and olfactory intensity remain to be explored. Similarly, it is unclear which specific visual features (e.g., grain, color, texture) contributed most to the observed effects. In addition, personal characteristics such as age, sex, affinity for nature, or olfactory sensitivity were not considered in the current study, and future research should examine differential responses based on such factors.

In summary, this study serves as an initial attempt to clarify the potentials and challenges of integrated sensory design involving wood-derived visual and olfactory stimuli. Future research should replicate these findings across more diverse environments and conditions, with the long-term goal of establishing a systematic, multisensory approach to wood-based spatial design.

## Figures and Tables

**Table 1:** Summary of experimental conditions.

ID	Wood-Derived Stimuli	Visual Stimuli: Olfactory Stimuli					
		Room A’s (Working Room’s) Stimulation			Room B’s (Resting room’s) Stimulation		
NN	None	Concrete-looking	:	None	Concrete-looking	:	None
HN-T	Only Hinoki scent	Concrete-looking	:	Hinoki oil	Concrete-looking	:	None
HN-R		Concrete-looking	:	None	Concrete-looking	:	Hinoki oil
NW-T	Only walnut panels	Walnut panels	:	None	Concrete-looking	:	None
NW-R		Concrete-looking	:	None	Walnut panels	:	None
HW-T	Both	Walnut panels	:	Hinoki oil	Concrete-looking	:	None
HW-R		Concrete-looking	:	None	Walnut panels	:	Hinoki oil

**Table 2:** Mean scores and statistical results for olfactory impression evaluation (*n* = 12).

Phase	Items	Mean ± SE			Pairwise Comparison
		NN	HN-T/R	HW-T/R	
After Task 1	(+3) good–bad (−3)	0.42 ± 0.29	1.33 ± 0.19	1.75 ± 0.22	HN-T > NN ** HW-T > NN ** HW-T > HN-T *
	(+3) like–dislike (−3)	0.42 ± 0.29	1.42 ± 0.23	1.75 ± 0.22	HN-T > NN ** HW-T > NN **
	(+3) familiar–unfamiliar (−3)	0.58 ± 0.31	1.42 ± 0.23	1.83 ± 0.24	
	(+3) settling–unsettling (−3)	0.75 ± 0.37	1.25 ± 0.25	1.67 ± 0.22	
	(+3) luxurious–simple (−3)	−0.67 ± 0.22	0.33 ± 0.22	0.5 ± 0.19	HN-T > NN ** HW-T > NN **
	(+3) mild–pungent (−3)	0.25 ± 0.25	1.00 ± 0.21	1.00 ± 0.28	
After Rest	(+3) good–bad (−3)	0.5 ± 0.29	1.75 ± 0.25	1.42 ± 0.26	HN-R > NN ** HW-R > NN **
	(+3) like–dislike (−3)	0.67 ± 0.26	1.75 ± 0.25	1.5 ± 0.29	HN-R > NN ** HW-R > NN *
	(+3) familiar–unfamiliar (−3)	0.92 ± 0.29	1.83 ± 0.21	1.58 ± 0.34	HN-R > NN **
	(+3) settling–unsettling (−3)	1.00 ± 0.29	1.83 ± 0.24	1.75 ± 0.35	
	(+3) luxurious–simple (−3)	−0.25 ± 0.25	0.67 ± 0.31	0.67 ± 0.19	HN-R > NN ** HW-R > NN **
	(+3) mild–pungent (−3)	0.67 ± 0.28	1.25 ± 0.22	1.08 ± 0.26	HW-R > NN *
After Task 2	(+3) good–bad (−3)	0.17 ± 0.11	1.42 ± 0.29	1.58 ± 0.26	HN-T > NN ** HW-T > NN **
	(+3) like–dislike (−3)	0.25 ± 0.18	1.42 ± 0.23	1.75 ± 0.3	HN-T > NN ** HW-T > NN **
	(+3) familiar–unfamiliar (−3)	0.42 ± 0.23	1.33 ± 0.26	1.75 ± 0.28	
	(+3) settling–unsettling (−3)	0.42 ± 0.23	1.42 ± 0.29	1.58 ± 0.26	HN-T > NN ** HW-T > NN **
	(+3) luxurious–simple (−3)	−0.58 ± 0.23	0.33 ± 0.31	0.58 ± 0.29	HN-T > NN ** HW-T > NN ** HW-T > HN-T *
	(+3) mild–pungent (−3)	0.25 ± 0.18	1.00 ± 0.17	0.92 ± 0.23	HN-T > NN * HW-T > NN *

Values are expressed as mean ± SE. Statistical comparisons were performed using Wilcoxon signed-rank tests with Holm correction. * *p* < 0.10, ** *p* < 0.05.

**Table 3:** Mean scores and statistical results for visual impression evaluation (*n* = 12).

Phase	Items	Mean ± SE			Pairwise Comparison
		NN	NW-T/R	HW-T/R	
After Task 1	(+3) like–dislike (−3)	0.58 ± 0.23	1.17 ± 0.21	1.33 ± 0.26	NW-T > NN * HW-T > NN *
	(+3) familiar–unfamiliar (−3)	0.58 ± 0.36	1.33 ± 0.19	1.17 ± 0.21	
	(+3) settling–unsettling (−3)	0.58 ± 0.29	1.33 ± 0.26	1.5 ± 0.23	NW-T > NN ** HW-T > NN **
	(+3) luxurious–simple (−3)	−0.58 ± 0.26	0.5 ± 0.15	0.42 ± 0.26	NW-T > NN ** HW-T > NN **
	(+3) spacious–small (−3)	−0.25 ± 0.22	0.33 ± 0.19	0.17 ± 0.24	
	(+3) natural–artificial (−3)	−0.5 ± 0.38	0.33 ± 0.26	0.17 ± 0.24	NW-T > NN * HW-T > NN *
After Rest	(+3) like–dislike (−3)	0.75 ± 0.22	1.33 ± 0.19	1.58 ± 0.26	NW-R > NN * HW-R > NN **
	(+3) familiar–unfamiliar (−3)	1.08 ± 0.31	1.42 ± 0.23	1.42 ± 0.31	
	(+3) settling–unsettling (−3)	1.17 ± 0.34	1.5 ± 0.26	1.5 ± 0.26	
	(+3) luxurious–simple (−3)	−0.33 ± 0.31	0.42 ± 0.23	0.42 ± 0.19	HW-R > NN *
	(+3) spacious–small (−3)	0.08 ± 0.26	0.5 ± 0.29	0.58 ± 0.38	
	(+3) natural–artificial (−3)	−0.33 ± 0.4	0.17 ± 0.32	0.17 ± 0.3	
After Task 2	(+3) like–dislike (−3)	0.83 ± 0.3	1.25 ± 0.25	1.5 ± 0.23	NW-T > NN * HW-T > NN *
	(+3) familiar–unfamiliar (−3)	0.92 ± 0.38	1.33 ± 0.26	1.25 ± 0.22	
	(+3) settling–unsettling (−3)	0.83 ± 0.42	1.58 ± 0.29	1.58 ± 0.26	
	(+3) luxurious–simple (−3)	−0.5 ± 0.29	0.58 ± 0.19	0.58 ± 0.26	NW-T > NN ** HW-T > NN **
	(+3) spacious–small (−3)	−0.33 ± 0.22	0.25 ± 0.22	0.42 ± 0.29	
	(+3) natural–artificial (−3)	−0.5 ± 0.4	0.33 ± 0.28	0.33 ± 0.26	NW-T > NN * HW-T > NN *

Values are expressed as mean ± SE. Statistical comparisons were performed using Wilcoxon signed-rank tests with Holm correction. * *p* < 0.10, ** *p* < 0.05.

## Data Availability

The data that support the study findings are available from the corresponding author upon reasonable request.
